# Antimicrobial activity, phytochemical characterization and gas chromatography-mass spectrometry analysis of *Aspilia pluriseta* Schweinf. extracts

**DOI:** 10.1016/j.heliyon.2020.e05195

**Published:** 2020-10-08

**Authors:** Sospeter N. Njeru, Jackson M. Muema

**Affiliations:** a Department of Biochemistry, School of Health Sciences, Kisii University, PO Box 408-40200, Kisii, Kenya; b Department of Biochemistry, Jomo Kenyatta University of Agriculture and Technology (JKUAT), PO Box 62000-00200, Nairobi, Kenya

**Keywords:** Tuberculosis, *Mycobacterium tuberculosis*, Drug resistance, Phytochemicals, Fatty acid alkyl esters, Alkanes, Medicinal/herbal plants, Traditional/folk medicine, Microbiology, Pharmaceutical science, Biochemistry, Infectious disease, Pharmacology, Alternative medicine, Laboratory medicine

## Abstract

*Aspilia pluriseta* is associated with various bioactivities, although with limited scientific justification. In this study, we evaluated the antimicrobial activity, and characterized the phytochemicals of root extracts of *A. pluriseta* aimed at validating its therapeutic potential. We used BACTEC MGIT™ 960 system to test for antitubercular activity, disc-diffusion together with the microdilution method to evaluate antimicrobial activities and qualitative phytochemical tests together with gas chromatography-mass spectrometry (GC-MS) analysis to determine the phytochemicals that associated with *A. pluriseta* extracts activity. We show that methanolic crude extract (at 1 g/mL) had high *Mycobacterium tuberculosis* (MTB) inhibitory activity (0 growth unit) and considerable potency against *Escherichia coli* (11.7 mm), *Staphylococcus aureus* (9.0 mm), and *Candida albicans* (7.7 mm). All the extract fractions exerted remarkable antimycobacterial activities with minimum inhibitory activity of between 6.26 – 25 μg/mL. The highest antimicrobial activity of petroleum ether and dichloromethane fraction was against *E. coli* at inhibition zone diameters of 8.3 mm, and 8.0 mm, respectively, while ethyl acetate fraction was against *S. aureus* with an inhibition zone of 8.7 mm. Methanolic fraction exhibited broad-spectrum activity against 87.5% of the tested microbes (inhibition zones 6.3–8.3 mm). Furthermore, we qualitatively detected terpenoids, alkaloids, and phenolics such as flavonoids, and anthraquinones in extract fractions. GC-MS analysis detected an abundance of fatty acid esters, 2-hydroxy-1-(hydroxymethyl) ethyl ester-hexadecanoic acid, and 2,3-dihydroxy propyl ester-octadecanoic acid and four alkanes. Taken together, we show that *A. pluriseta* extract fractions (especially ethyl acetate and methanolic fractions) have strong selective antitubercular activity, and thus, we scientifically validate the use of *A. pluriseta* as a potential source for the discovery of novel antitubercular agents.

## 1 Introduction

Infectious diseases are a primary cause of global human and animal mortality, which is further aggravated by frequent emergence and re-emergence of opportunistic infections [1]. However, one of the major global health challenges is attributed to tuberculosis. Tuberculosis (TB) is the leading cause of mortalities from a single infectious agent, which claimed the lives of over 1 million people, besides making an additional over 10 million people ill in 2018 [2,3]. In humans, TB is an airborne infection primarily caused by MTB [3, 4]. MTB thrives in the hostile environment of the human lungs, despite a sustained immunological onslaught of the host that prevents the growth of nearly all other bacteria [3, 5]. MTB effectively survives host defenses because of a highly impermeable cellular envelope that covers it. The mycobacterial cell envelope is a complex heteropolymer composed of peptidoglycan covalently attached to arabinogalactan terminated by mycolic acids, specific to mycobacteria. Also, MTB can manipulate the host immunological defense mechanisms to foster its survival in a harsh environment [5].

Effective management of MTB is hampered by a number of factors such as; (1) The widespread development of drug resistance (for example, multi-drug resistant TB (MDR-TB) which does not respond to isoniazid and rifampicin, and extensively drug-resistant TB (XDR-TB) which is resistant to isoniazid, rifampin, plus fluoroquinolones and one of the injectable second-line drugs such as amikacin, kanamycin, or capreomycin used in MDR-TB treatment regimens) [6,7], (2) The manifestation of asymptomatic latent TB infection in nearly a fourth of the global population, (3) Expensive and lengthy treatment regime, (4) Drug toxicity and associated adverse effects, and (5) Slow phase discovery of new antitubercular agents among other factors [3, 8, 9, 10]. Therefore, it is clear that further discovery and development of novel complementary treatment options are needed for improved TB treatment and control. This will significantly contribute to the World Health Organization (WHO) EndTB strategy [3, 8, 11].

Besides TB, there are other Gram-positive, Gram-negative, and fungal pathogens that have acquired noxious drug resistance. These pathogens are often responsible for the hospital- and community-acquired infections including and not limited to methicillin-resistant *Staphylococcus aureus* (MRSA), *Klebsiella pneumoniae,* fluoroquinolone-resistant *Pseudomonas aeruginosa*, *Escherichia coli,* among others [12]. Such emerging drug-resistant pathogenic strains are usually not sensitive to the first-line of antimicrobial therapy, thus forcing the use of a second- and third-line treatment option. Besides narrowing the available treatment options, the adoption of second- and third-line classes of drugs is often associated with severe adverse effects [13]. This further highlights the need for concerted effort in prospecting for novel formulations that are active against emerging, re-emerging, and drug-resistant pathogenic strain.

Although it is widely accepted that drug discovery is an expensive and time-consuming venture, exploration of certain plant species as an alternative source of pharmaceutical molecules, guided by traditional/indigenous knowledge has been very promising [14, 15, 16, 17, 18, 19]. The pharmacological activities of plant species are attributed to their phytochemicals such as terpenoids, saponins, flavonoids, glycosides, alkaloids, steroids, among others [19, 20]. The traditional/folk knowledge on medicinal plants has been heavily relied on since ancient times in search of new pharmaceutical molecules, antimicrobials, chemotherapeutics, as well as antitubercular agents [20, 21, 22, 23, 24]. Folk medicine has been applied to treat a wide range of diseases and infections, and reports are suggesting that plant-derived compounds exhibit fewer side effects, are less toxic, have low propensity to develop resistance, and are associated with improved efficacy [18, 20]. Herbal medicine is additionally prevalent in under-developed and developing countries where infectious diseases are common, compounded by poverty, poor hygiene and sanitation, and inaccessibility or high cost of good healthcare [25, 26]. In fact, it is estimated that 80% of the under-developed and developing world population rely on plant-derived remedies for alleviating various ailments [19, 27, 28, 29, 30, 31]. This fact is reinforced by the findings that plants are a rich source of bioactive molecules as efficacious as synthetic pharmaceutics or that can either be chemically modified to enhance their potency or act as templates for new pharmacophores [19, 32, 33, 34, 35]. Furthermore, plant extracts have multitargeting capacity since they contain multiple small biomolecules in low concentration, but showing synergistic and additive effects. In theory, plant extracts function in a 'polypharmacology' paradigm, entailing the use of a single product against multiple targets. This paradigm is touted as a game-changer against drug-resistant microbial strains, besides being associated with low toxicity and low side effects [3]. Therefore, the integration of traditional medicine into modern medicine is under serious consideration so as to ease drug resistance challenges and provide an alternative source for affordable, safe, and effective drugs [36, 37]. However, implementation of such herbal derived medicines requires in-depth scrutiny of their efficacy and identification of specific bioactive constituents [17, 30, 31, 38, 39]. This would provide rational scientific validation and justification for utilization and possible integration into conventional medicine, a measure that could strongly contribute to affordable, improved public health [19].

In this study, we used *Aspilia pluriseta* Schweinf. (*Asteraceae*) to investigate its potential antimicrobial activity and characterize the phytochemicals responsible for the bioactivity. *A. pluriseta* is locally known as Muuti (in Mbeere, Embu, and Kikuyu), Wuti (in Kamba), Ol-oiyabase (in Maasai), and Shilambila (in Luhya) communities of Kenya [40]. This plant is widely spread in East, Central, and Southern Africa, especially in open woodlands and grasslands [41, 42]. In East Africa (especially Kenya, Rwanda, and Uganda), the plant has been documented to manage and treat cough, stomach infections, burns, bruises, lacerations, wounds, pimples, ears-, eye-, nose infections, kwashiorkor, fever, worms, and diabetes mellitus with little or no scientific validation [40, 42, 43, 44, 45, 46, 47]. There are reports on antiviral activity [42], molluscicidal activity [48], complement modulating activity [49], antihelmintic activity [50], antimalarial, and hypoglycaemic activities [40, 47, 51] of *A. pluriseta*. However, the scientific evidence of its pharmacological activity against medically important bacteria, especially MTB, is at its early stages. As part of our continuous research efforts to discover novel, potent, antitubercular agents from Kenyans ethnobotanicals, in this study we describe the antitubercular activity of *A. pluriseta* solvent extract fractions. Our findings demonstrate that *A. pluriseta* extract fractions (especially ethyl acetate and methanolic fractions) have remarkable selective antitubercular activity, which is partly if not exclusively associated with phytochemicals such as terpenoids, phenolics, alkaloids, fatty acid alkyl esters detected in the extract fractions.

## 2 Methods

### 2.1 Plant material collection

We used the ethnopharmacological approach to identify the plant used in this study. The information on its herbal use and preparation among the Mbeere community of Embu County, Kenya, was gleaned from community herbal practitioners and further confirmed from documentation by Riley and Brokensha (1988) in *The Mbeere in Kenya (ii), Botanical identity and use* [52]. We collected the plant root materials in an open community field. The plant is not among the endangered species, and therefore no prior permission was sought before sample collection. The sampling was carried out within 0°46′27.0″S 37°40′54.9″E; -0.774156, 37.681908 of GPS co-ordinates. A botanist authenticated plant sample identity at Egerton University, Kenya, where voucher specimen number NSN2 was given and deposited.

### 2.2 Extraction and fractionation of active ingredients

The root samples were mechanically size-reduced, air-dried in the dark at 23 ± 2 °C to a constant weight, then ground into a fine powder using an electric miller (Retsch SR 200, Haan, Germany). In order to mimic the traditional preparation method, a portion of the sample powder (50 g) was subjected to cold extraction in distilled water with occasional shaking, after which the extract was lyophilized. A similar portion was macerated twice in 200 mL methanol (Sigma Aldrich, St. Louis, USA) for 48 h, pooled and filtered using Whatmann 1 filter paper. Excess methanol was evaporated from the filtrate using a rotor evaporator (Laborota 4000 efficient, Heidolph, Germany) and the extract stored at -20 °C until use. Fractionation of the *A. pluriseta* extract was performed using organic solvents of increasing polarity. Fifty grams of root powder was macerated in 200 mL of petro ether with intermittent shaking for 48 h. Subsequently, the material was filtered through Whatman number-1 filter paper. The residue was additionally re-extracted using the same fresh solvent for 48 h, and after that, the two filtrates pooled together. The resulting marc was air-dried and further extracted with dichloromethane solvent followed by ethyl acetate, and methanol solvent, using the same procedure carried out for petroleum ether. Organic crude extract and solvent fractions were concentrated and reconstituted into appropriate stock solution with 100% dimethyl sulfoxide (DMSO) but diluted appropriately so that the final DMSO in the test sample is 1% DMSO. Water crude extract was reconstituted in physiological saline, which served as its negative control.

### 2.3 Antimicrobial activity

#### 2.3.1 Test microorganisms

All the test microorganisms were sourced from Kenya Medical Research Institute (KEMRI), Nairobi. These included; one acid-fast *Mycobacterium tuberculosis* strain H37Rv (ATCC 27294), one Gram-positive; *Staphylococcus aureus* (ATTC 25923) strain and Methicillin-resistant *Staphylococcus aureus* strain (clinical isolate), five Gram-negative bacteria; *Escherichia coli* (ATTC 25922), *Pseudomonas aeruginosa* (ATCC 27853), *Klebsiella pneumoniae* (clinical isolate), *Salmonella typhi* (clinical isolate) and *Shigella sonnei* (clinical isolate), and two fungi; *Candida albicans* (ATTC 90028), *Cryptococcus neoformans* (ATTC 66031).

#### 2.3.2 Antimycobacterial activity

MTB was revived in Lowenstein Jensen slants under previously adopted standard conditions [53, 54] and later subjected to BACTEC MGIT 960 system (BD Biosciences, New York, USA) for antitubercular activity assays [55, 56]. BACTEC MGIT 960 system is a fully automated, high volume, a non-radiometric instrument that undertakes continuous monitoring of culture growth. Growth supplement (0.8 mL) containing a combination of oleic acid, dextrose, bovine albumin, and catalase was added to five 7 mL BBL™ MGIT™ tubes labeled GC (growth control), STR (streptomycin), INH (isoniazid), RIF (rifampicin), EMB (ethambutol) to provide essential substrates for the rapid growth of MTB. MTB suspension in 0.1 mL Middlebrook 7H9 broth adjusted to 0.5 McFarland standard with 10 mL sterile physiological saline was aseptically transferred into each BBL™ MGIT™ tube and incubated at 37 °C. One hundred microliters of BBL™ MGIT™ SIRE (streptomycin, isoniazid, rifampicin, ethambutol) prepared aseptically following the manufacturers' instructions were added into corresponding labeled BBL™ MGIT™ tube followed by addition of 0.5 mL of 1% MTB suspension. Streptomycin at 1.0 μg/mL, rifampicin at 1.0 μg/mL, ethambutol at 5.0 μg/mL, and isoniazid at 0.1 μg/mL were used as the positive controls whereas 1% DMSO (for solvent extract, and solvent extract fractions), and sterile physiological saline (for water extract) were used as negative controls. The protocol was repeated using crude extracts at 1.0 g/mL (for screening purposes) in place of SIRE and solvent fractions tested at concentrations ranging from 50 to 6.25 μg/mL for petroleum ether, dichloromethane, and methanol, and 25 to 3.125 μg/mL for ethyl acetate to determine the MTB minimum inhibitory concentration (MIC).

#### 2.3.3 Disc diffusion test

To evaluate the general antimicrobial activity of *A. pluriseta* crude and solvent fractions extracts at various specified concentrations, we used the modified disc diffusion method [57, 58, 59, 60, 61]. A fresh microbial inoculum was made by suspending activated colonies in physiological saline. The bacterial and fungal suspensions were adjusted to 1.5 × 10^6^ CFU/mL using 0.5 McFarland turbidity standard and aseptically inoculated onto Muller Hinton agar (MHA) and Sabouraud Dextrose agar (SDA) plates, respectively. Sterilized Whatmann 1 filter paper discs (diameter 6 mm) were impregnated with 10 μL of stock extract solutions (1.0 g/mL crude methanol and water extracts, 500 μg/mL for petroleum ether, dichloromethane, methanolic fractions, and 250 μg/mL for ethyl acetate fraction (inadequate amounts)). Three standard drugs were used as antibiotics positive controls; Oxacillin at 10 μg/disc (Oxoid Ltd, Tokyo, Japan) for Gram-positive bacteria, Gentamycin at 10 μg/disc (Oxoid Ltd, Tokyo, Japan) for Gram-negative bacteria, and Nystatin at 100 μg/disc (Oxoid Ltd, Tokyo, Japan) for all fungi. Whatmann filter paper discs loaded with 10 μL of 1% DMSO (for solvent extract, and solvent extract fractions), and 10 μL of sterile physiological saline (for water crude extract) served as negative controls. Air-dried discs were carefully placed on the agar plates at equidistance points using sterile forceps, including both positive antibiotic control and negative control discs into each plate. Subsequently, the plates were initially incubated at 4 °C for 2 h to allow pre-diffusion of extracts into media and incubated at 37 °C for 24 h. Antimicrobial activity was assessed in triplicates by measuring the size of the inhibition zone to the nearest mm. Fractions exhibiting strong antimicrobial inhibitory potential were considered for further MIC and minimum microbicidal concentration analysis (MMC) determination [62].

#### 2.3.4 Determination of MIC and MMC

The MIC and MMC of *A. pluriseta* solvent fractions were analyzed as previously described [36, 57, 60, 61, 63]. Briefly, 50 μL of varying fraction concentrations (3.9–500 μg/mL petroleum ether, dichloromethane, and methanol; 1.95–250 μg/mL ethyl acetate) were added into 100 μL of nutrient broth held in a sterile 96-well plate followed by addition of 50 μL test organisms adjusted to 0.5 McFarland standard. All concentrations were tested in triplicates at 37 °C for 24 h. A negative control containing 1% DMSO in nutrient broth was included in column 11, while column 12 checked the capacity of the media to support the growth of the test organism. In order to evaluate the microbial growth in each well, 40 μL of 0.2 mg/mL *p*-iodonitrotetrazolium chloride (INT, Sigma) were added and incubated for 30 min. Formation of a pink-red color depicted growth while persistent clear coloration denoted growth inhibition. The lowest solvent fraction concentration that exhibited color change was considered as the MIC. MMC was determined by aseptically streaking a loopful of broth from wells that exhibited no color change onto sterile nutrient agar and SDA for bacteria and fungi, respectively, and thereafter incubated at 37 °C for 24 h. The lowest concentration that exhibited no growth was considered as the MMC [64].

### 2.4 Phytochemical analysis using GC-MS

We performed a preliminary screening for the presence of various phytochemicals such as alkaloids, terpenoids, phenolics such as flavonoids, and anthraquinones qualitatively as previously reported by us and others [4, 20, 36, 65]. Additionally, we undertook a GC-MS analysis of the methanolic extract fraction since it exhibited a broad-spectrum activity. An aliquot of the methanolic fraction (1.3 mg) was dissolved in 1 mL dichloromethane and analyzed with an Agilent Technologies 7890A gas chromatography coupled with a 5975C mass spectrometer in full scan mode (EI, 70 eV, Agilent, Palo Alto, CA). The system was equipped with an HP-5 MS low bleed capillary column (30 m × 0.25 mm i.d., 0.25 μm film thickness (J&W, Folsom, CA, USA)). An injection volume of 1 μL was subjected to a splitless mode during analysis, with helium used as the carrier medium at a constant flow rate of 1.25 mL/min. The oven temperature was maintained at 35 °C for 5 min, then programmed to increase at 10 °C/min to 280 °C and held at this temperature for 10.5 min. The obtained compound profiles were identified by comparing the corresponding reference retention indices and mass spectral in databases (NIST 05, NIST 08, Adams, and chemical).

### 2.5 Data analyses

The data was analyzed using Analysis of variance (ANOVA) using R (version 3.5.1) with Tukey HSD post-hoc. A *p-*value of less than 0.05 was considered statistically significance. Values were expressed as mean ± SEM of experimental replicates.

## 3 Results

### 3.1 Screening for general antimicrobial activities of *A. pluriseta* crude extracts

In order to mimic the traditional preparation of *A. pluriseta* herbal medicine, we initially assayed for the general bioactivity of the crude water and methanol extracts. Using a BACTEC MGIT™ 960 system (BD, New York, USA) to assay for antimycobacterial activity, we found that the water crude extract had no antituberculous activity (400 growth unit (GU)), equivalent to GU of the negative control. Interestingly, the methanolic crude extract exhibited high inhibitory activity against MTB similar to SIRE positive control (0 GU; Table 1).

**Table 1:** Screening for the antimycobacterial activity of *A. pluriseta* crude extracts.

Sample	Solvent	GU	NR/S
*A. pluriseta*	Water	400	NR
	Methanol	0	S
	SIRE	0	S
	GC	400	NR

Water and methanol crude extract at 1 g/mL; SIRE: Positive control of streptomycin at 1.0 μg/mL, isoniazid at 0.5 μg/mL, rifampicin at 1.0 μg/mL and ethambutol at 5.0 μg/mL; GC: Growth control acting as a negative control of media treated with 1% DMSO (methanolic crude extract) or physiological saline (water crude extract); NR: Non-responsive; S: Sensitive.

Further, we screened for general antimicrobial activity by disc diffusion method against representative Gram-positive bacteria (*S. aureus*), Gram-negative bacteria (*E. coli*), and fungi (*C. albicans*). Our results demonstrated a significant difference in antimicrobial activities of tested extracts against test microbes relative to controls (ANOVA, *S. aureus*; F_(3,8)_ = 160.1, *p* < 0.001; *E. coli*, F_(3,8)_ = 53.67, *p* < 0.001; *C. albicans*, F_(3,8)_ = 72.67, *p* < 0.001). The water extract exhibited low general antimicrobial activity in all tested cases (zone of inhibition <10 mm), while the methanolic crude extract gave moderate but broad-spectrum activity, with the highest inhibition of 11.7 mm against *E. coli* (Table 2).

**Table 2:** Screening for general antimicrobial activity of *A. pluriseta* crude extracts.

Sample	Extract	The diameter of zone of inhibition (mm)		
		*S. aureus*	*E. coli*	*C. albicans*
*A. pluriseta*	Water	6.7 ± 0.3^ab^	7 ± 0.6 ^ab^	6.0 ± 0^ab^
	Methanol	9.0 ± 0.6 ^ab^	11.7 ± 0.3^b^	7.7 ± 0.3 ^ab^
	Positive control	24.0 ± 1.3	22.0 ± 0	16.3 ± 0.9
	Negative control	0	0	0

Water and methanol crude extract at 10 × 10^4^ μg; Positive control (Oxacillin 10 μg/disc for Gram-positive, Gentamycin 10 μg/disc for Gram-negative bacteria and Nystatin 100 μg/disc for fungi); Negative control (Discs loaded with 10 μL of 1% DMSO (for methanol extract) or physiological saline (for water extract)); n = 3; Values = Mean ± SEM. Values followed by similar superscript letters are not significantly different from each other (ANOVA - Tukey's post-hoc multiple comparisons, *P* < 0.05).

Even though the crude extract concentrations were in the range of 10^4^ times higher than the standard antibiotic controls, methanolic crude extract showed a remarkable antitubercular activity, that compared with the activity of the positive control. Since the active components in the crude extract could have comprised only a fraction of the total extract used, we reasoned that further purification would allow enrichment of active molecules in extract fractions. Therefore, we hypothesized that extract solvent fractionation would result in enhanced activity, and possibly at a lower concentration.

### 3.2 Antimycobacterial activities of *A. pluriseta* solvent extract fractions

To test whether fractionation of *A. pluriseta* could lead to improved antimycobacterial activity, as well as determine the MIC of different fractions, we used the BACTEC MGIT 960 system. If the GU of the extract fraction-containing tubes was greater than 100 when the GU of the growth control was 400, we defined the results as non-responsive. However, if the GU values of the extract fraction-containing tubes were ≤100, the results were considered susceptible, and the concentration of that tube was used to define the MIC [55, 56]. Our results revealed the most robust activity against MTB by more polar ethyl acetate (EA) fraction (MIC 6.25 μg/mL) followed by methanolic (MeOH) fraction (12.5 μg/mL). The less polar dichloromethane (DCM) and petroleum ether (PE) fractions had a MIC of 25 μg/mL, with DCM fraction inhibiting MTB growth in a dose-dependent manner (Table 3). For EA and MeOH fractions, concentrations ≥6.25 and 12.5 μg/mL, respectively, completely inhibited MTB growth, an observation comparable to a positive control (SIRE). These results confirmed our hypothesis that *A. pluriseta* fractionation would lead to robust antitubercular activity, and at a lower concentration. Further, the results are in agreement with other studies that have reported polar solvent extract fractions usually have higher activity than less polar fractions [66, 67, 68].

**Table 3:** Antimycobacterial activity of *A. pluriseta* solvent extract fractions.

Plant	Fraction	Concentration μg/mL	GU	NR/S	MIC (μg/mL)
*A. pluriseta*	PE	50	0	S	25
		25	0	S	
		12.5	400	NR	
		GC	400	NR	
		SIRE	0	S	
	DCM	50	0	S	25
		25	3	S	
		12.5	132	S	
		GC	400	NR	
		SIRE	0	S	
	EA	25	0	S	6.25
		12.5	0	S	
		6.25	0	S	
		GC	400	NR	
		SIRE	0	S	
	MeOH	50	0	S	12.5
		25	0	S	
		12.5	0	S	
		GC	400	NR	
		SIRE	0	S	

PE: Petroleum ether fraction; DCM: Dichloromethane fraction; EA: Ethyl acetate fraction; MeOH: Methanol fraction; SIRE: Positive control of streptomycin at 1.0 μg/mL, isoniazid at 0.5 μg/mL, rifampicin at 1.0 μg/mL and ethambutol at 5.0 μg/ml; GU: Growth unit; GC: Growth control as the negative control of media treated with 1% DMSO; **N**R: Non-responsive; S: Sensitive.

### 3.3 General antimicrobial activities, MIC and MMC of *A. pluriseta* extract fractions

With crude solvent extract having demonstrated moderate broad-spectrum antimicrobial activity, we reasoned that solvent fractionation might yield improved antimicrobial activity. However, fractionation of root extract with solvents of increasing polarities resulted in attenuated but broad-spectrum mild antimicrobial activity, with all fractions giving inhibitory zones of less than 10 mm (Table 4). The best activity (though still a weak one) by petroleum ether fraction was against *E. coli* (zone of inhibition 8.3 mm), dichloromethane against *E. coli* (8.0 mm), ethyl acetate against *S. aureus* (8.7 mm) and methanolic against *S. aureus* (8.3 mm), *C. albicans* (8.3 mm) and against *E. coli* (8.0 mm) (Table 4). EA fraction showed a weak growth inhibition against *C. neoformans* (zone of inhibition 6.7 mm), while MeOH fraction weakly inhibited *K. pneumonia* (zone of inhibition 7.0 mm). Generally, the methanolic extract fraction exhibited significant broad spectrum activity against seven of the eight tested microorganisms (ANOVA; *S. aureus*, F_(5,12)_ = 2267, *p* < 0.001; MRSA, F_(4,10)_ = 129.7, *p* < 0.001; PA, F_(5,12)_ = 2464, *p* < 0.001; *E. coli*, F_(5,12)_ = 248.3, *p* < 0.001; KP, F_(5,12)_ = 922.1, *p* < 0.001; S.S, F_(5,12)_ = 507, *p* < 0.001; ST, F_(5,12)_ = 143.7; *p* < 0.001; CN, F_(3,8)_ = 1849; *p* < 0.001; CA, F_(5,12)_ = 302.7; *p* < 0.001). Equally, relative to the negative controls, all the extract fractions significantly inhibited the growth of tested microorganisms (*p* < 0.001) but in a manner less comparable to the positive control.

**Table 4:** Antimicrobial activity of *A. pluriseta* solvent extract fractions.

Fractions	The diameter of zone of inhibition (mm)								
	Gram-positive		Gram-negative					Fungi	
	SA	MRSA	PA	EC	KP	SS	ST	CA	CN
PE	7.7 ± 0.3^ab^	NT	0^b^	8.3 ± 0.3^ab^	0^b^	0^b^	0^b^	7.7 ± 0.3^ab^	NT
DCM	7.7 ± 0.3^ab^	0^b^	0^b^	8.0±0^ab^	0^b^	7.7 ± 0.3^ab^	0^b^	7.3 ± 0.3^ab^	0^b^
EA	8.7 ± 0.3^ab^	0^b^	0^b^	0^b^	0^b^	0^b^	0^b^	0^b^	6.7 ± 0.3^ab^
MeOH	8.3 ± 0.3^ab^	6.3 ± 0.3^ab^	0^b^	8.0±0^ab^	7.0±0^ab^	8.0±0^ab^	6.7 ± 0.3^ab^	8.3 ± 0.3^ab^	NT
PC	33.7 ± 0.3	24.3 ± 0.3	23.7 ± 0.6	15 ± 0	15.7 ± 0.3	19.7 ± 0.6	21.3 ± 0.3	16.3 ± 0.3	20.3 ± 0.3
NC	0	0	0	0	0	0	0	0	0

PE: Petroleum ether fraction at 5 μg/disc; DCM: Dichloromethane fraction at 5 μg/disc; EA: Ethyl acetate fraction at 2.5 μg/disc; MeOH: Methanol fraction at 5 μg/disc; PA: *Pseudomonas aeruginosa*; EC: *Escherichia coli*; SA: *Staphylococcus aureus*; KP: *Klebsiella pneumoniae*; MRSA: *Methicillin Resistant Staphylococcus aureus*; SS: *Shigella sonnei*; ST: *Salmonella typhi*; CA: *Candida albicans*; CN: *Cryptococcus neoformans*; PC: Positive control (Oxacillin 10 μg/disc and Gentamycin 10 μg/disc for Gram positive and Gram negative bacteria respectively. Nystatin 100 μg/disc for fungi); NC: Negative control (Discs loaded with 10 μl of 1% DMSO); *n* = 3; values = Mean ± SEM; Values followed by similar superscript letters are not significantly different from each other (ANOVA - Tukey's post-hoc multiple comparisons, P < 0.05).

Further, we tested for the MIC and MMC concentrations of the solvent extract fractions (Table 5), and we established that petroleum ether fraction against *E. coli* had a MIC of 250 μg/mL while methanolic fraction against *S. aureus* had a MIC of 125 μg/mL. In all other cases, the MIC was greater than 500 μg/mL indicating that the extract fractions were bacteriostatic in action.

**Table 5:** MIC and MBC of *A. pluriseta* solvent extract fractions.

Fractions	MIC (μg/ml)								
	Gram-positive		Gram-negative					Fungi	
	SA	MRSA	PA	EC	KP	SS	ST	CA	CN
PE	-	-	-	250	-	-	-	-	-
DCM	-	-	-	-	-	-	-	-	-
EA	-	-	-	-	-	-	-	-	-
MOH	125	-	-	-	-	-	-	-	-

PE: Petroleum ether fraction; DCM: Dichloromethane fraction; EA: Ethyl acetate fraction; MOH: Methanol fraction; PA: *Pseudomonas aeruginosa*; EC: *Escherichia coli*; SA: *Staphylococcus aureus*; KP: *Klebsiella pneumoniae*; MRSA: *Methicillin-resistant Staphylococcus aureus*; SS: *Shigella sonnei*; ST: *Salmonella typhi*; CA: *Candida albicans*; CN: *Cryptococcus neoformans*; - indicates the MIC is >500 μg/mL; MMC in all cases were >500 μg/mL.

Therefore, the general antimicrobial activity results were counterintuitive, considering the fractions yielded robust antimycobacterial activity. This would suggest that the active principles in the extract fractions are, to some extent, specific to acid-fast bacteria. This would be very important for the discovery of selective antitubercular leads, as previously reported [69, 70].

### 3.4 Phytochemical analysis

The preliminary examination of *A. pluriseta* solvent extract fractions pointed to the presence of different phytoconstituents such as terpenoids, alkaloids, and phenolics such as flavonoids, and anthraquinones (Table 6). We speculate that these are the bioactive constituents that may be responsible for the bioactivity demonstrated by these extract fractions.

**Table 6:** Phytochemical results of *A. pluriseta* solvent fraction extracts.

Extract Fraction	V-Ts	A-F	MK-A	D-A	F-P
Petrol ether	+++	-	-	+	-
Dichloromethane	+++	+	+	++	++
Ethyl acetate	+	+	+	-	+
Methanol	+++	-	-	-	+

**V-T,** Vanillin test for terpenoids; **A-F,** Ammonia test for Flavonoids; **MK-A,** Methanolic Potassium hydroxide test for Anthraquinones; **D-A,** Dragendorff test for Alkaloids; **F-P,** Ferric Chloride test for Phenols; **-,** Absent phytochemicals**; +,** Low concentration of phytochemicals; **++,** Medium concentration; **+++,** High concentration of phytochemicals.

**Figure 1:**
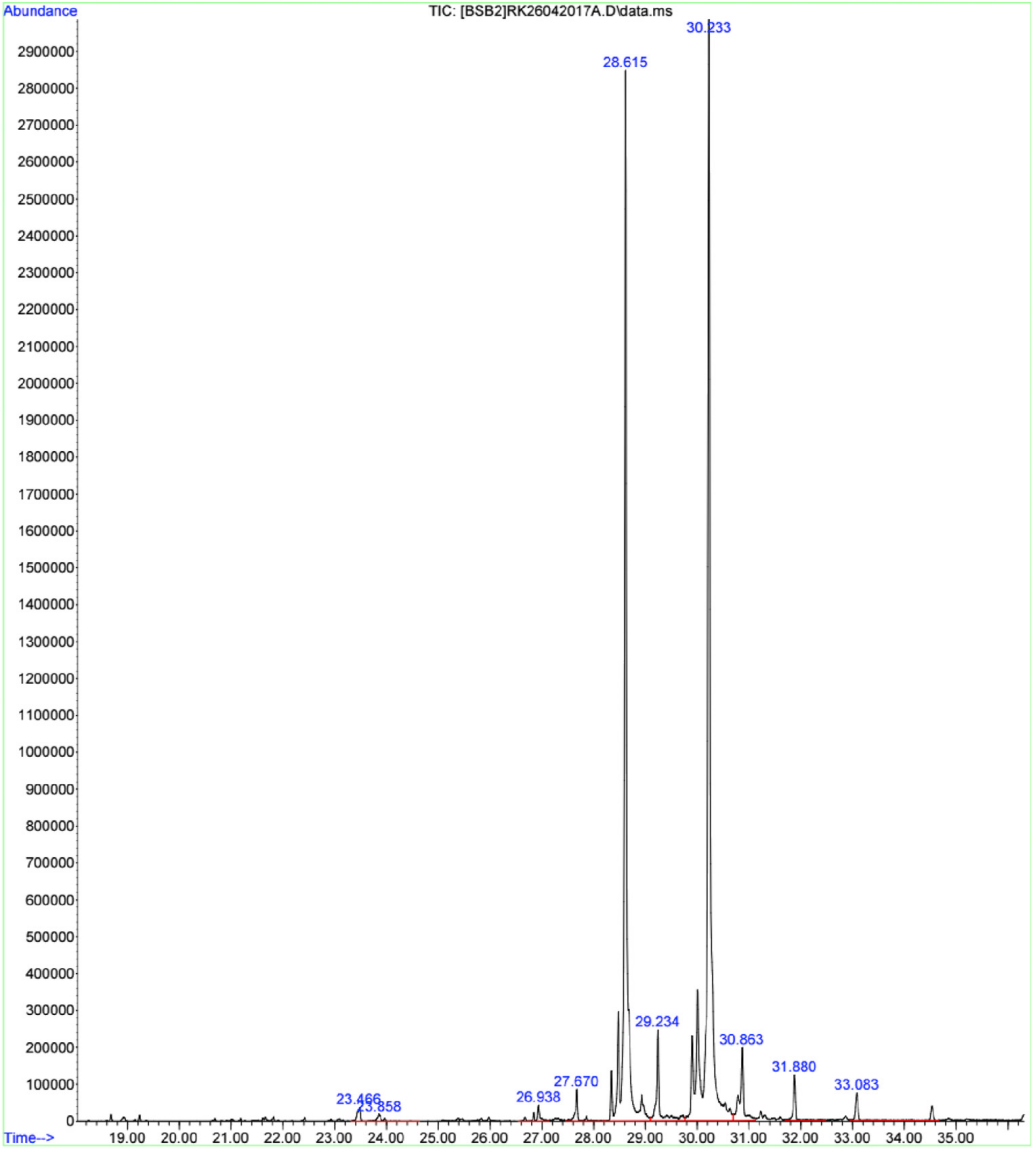
GC-MS analysis spectrum of *A. pluriseta* methanolic extract fraction. The spectrum highlights the compound abundance and separation based on mass fragmentation and retention times.

**Figure 2:**
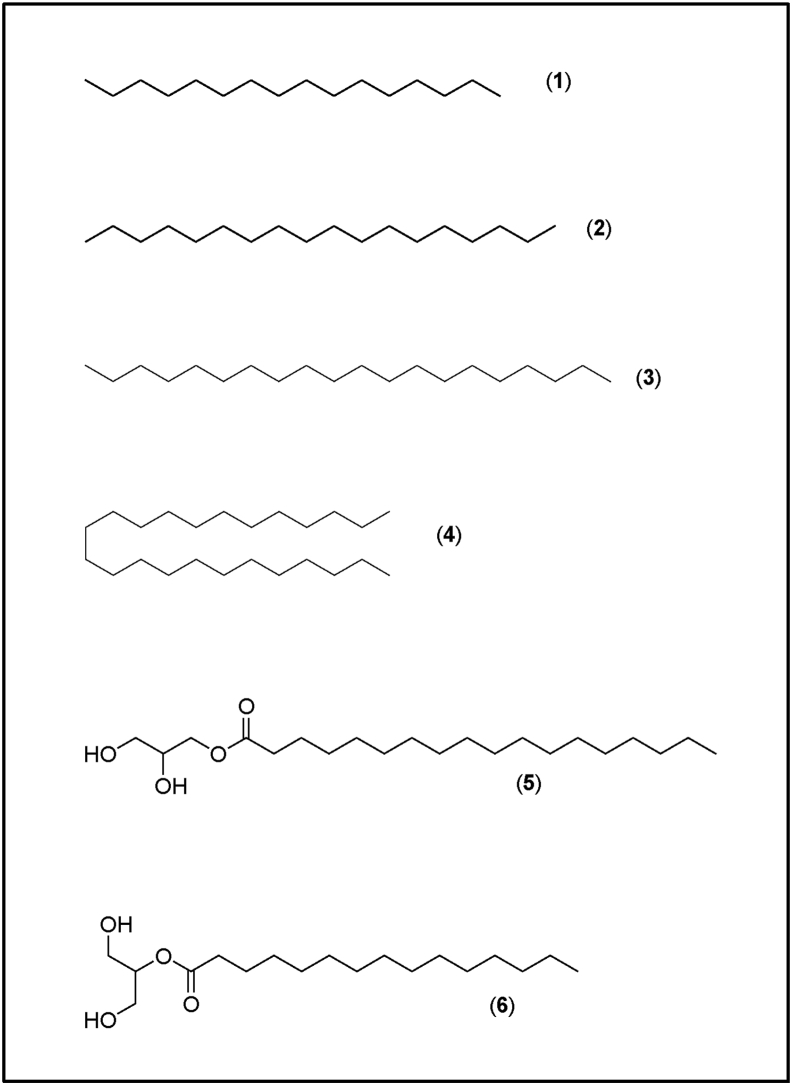
Compounds identified in *A. pluriseta* methanolic extract fraction using GC-MS. The chemical structures of the identified compounds (1) Hexadecane, (2) Octadecane, (3) Eicosane, (4) 2-hydroxy-1-(hydroxymethyl)ethyl ester-hexadecanoic acid, (5) Tetracosane, and (6) 2,3-dihydroxy propyl ester-octadecanoic acid.

Methanolic extract fraction exhibited broad spectrum activity (inhibiting the growth of Gram-negative, Gram-positive, acid-fast bacteria, and fungi (Tables 3 and 4)). Additionally, it was easier to get more materials from this extract fraction for further analysis. Therefore, we subjected this fraction to GC-MS analysis to identify the specific bioactive compounds that could be partly associated with the activity of this fraction (Table 7; Figures 1 and 2). We detected six compounds from the extract fraction, four alkanes, and two fatty acid esters. Based on the mass spectral library databases, these compounds were tentatively identified as hexadecane, octadecane, eicosane, 2-hydroxy-1-(hydroxymethyl) ethyl ester-hexadecanoic acid, tetracosane, and 2,3-dihydroxy propyl ester-octadecanoic acid (Table 7; Figure 2). The fatty acid esters represented by peaks at retention times 28.61 min (2-hydroxy-1-(hydroxymethyl) ethyl ester-hexadecanoic acid) and 30.23 min (2,3-dihydroxy propyl ester-octadecanoic acid) were the most abundant compounds detected (Figure 1), and it is highly possible that on their own or in combination with other secondary metabolites contributed wholly or in part to the bioactivity of this extract fraction.

**Table 7:** Compounds identified in *A. pluriseta* methanolic extract fraction using GC-MS.

Peak no.	Rt (min)	Compound name	Class
1	26. 84	Hexadecane	Alkane
2	27.67	Octadecane	Alkane
3	27.68	Eicosane	Alkane
4	28.61	2-hydroxy-1-(hydroxymethyl)ethyl ester-hexadecanoic acid	Fatty acid ester
5	29.24	Tetracosane	Alkane
6	30.23	2,3-dihydroxypropyl ester-octadecanoic acid	Fatty acid ester

Rt: Retention time in minutes; The data presented in the table above shows the peak number, corresponding retention time, compound identities, and their classification.

## 4 Discussion

Increasing demand for effective antimicrobials to lessen antimicrobial drug resistance burden and accelerate prompt prevention and treatment of microbial infections necessitates the discovery of new pharmaceutical molecules. This calls for a collaborative approach involving the herbal practitioners and scientific community in search of pharmaceutical molecules from traditionally-claimed active plants and scientifically validating their bioactivity. This comes at a time when most pharmaceutical industries seem reluctant and/or slow to develop novel antimicrobial agents, prompting an overreliance on the limited available antibiotics. This has consequently led to the emergence of superbugs insensitive to available antibiotic regimes [71, 72]. Flashing back to before the advent of first-generation antibiotics in 1928, humans were using and are still using herbal preparations to manage, treat, and cure various ailments. Herbal medicine thus provides a solid foundation for the discovery of new agents against these health-threatening pathogens [73, 74].

The current study was motivated by an ethno-based claim by the Ambeere residents and herbalists from Embu county - Kenya that, *A*. *pluriseta* root extracts are used in the management of 'strong' coughs and complicated respiratory tract infections. We hypothesized that the alluded to 'strong' cough and complicated respiratory tract infection represented TB. We therefore sought to investigate the antitubercular and general antimicrobial activities of this plant, as well as identify the bioactive compounds therein that could be responsible for the bioactivity.

The traditional-based approach of preparing the extracts using water yielded very low antitubercular and antimicrobial activities, while the crude methanolic extract exhibited a broad-spectrum antimicrobial activity against acid-fast, Gram-positive, Gram-negative, and fungi. This could point to a possible implication of differential solubility of various plant compounds in solvents of different polarities. It is established that solvent polarity affects the qualitative and quantitative composition of bioactive compounds enriched in various solvents, and that methanol is a better and more powerful extractant compared to water [66, 67, 68]. With methanolic crude extract giving a robust antitubercular activity, the next question we asked ourselves was whether solvent fractionation would give us more fractions with potent activity, and at a lower concentration (from 1 g/mL to between 50-6.25 μg/mL; Tables 1 and 3). Compared to the positive control, the concentration of the crude methanolic extract was 10^4^-fold higher. We reasoned that the active compound(s) in the crude methanolic extract comprised only a small fraction of the crude extract and if purified and isolated, might work at a lower concentration, or even more potently than the standard antibiotic used. To test this assumption, we fractionated the plant sample by organic solvents of increasing concentration. Interestingly, all solvent extract fractions had a strong antitubercular activity with MIC ranging between 25 and 6.25 μg/mL that yielded similar inhibitory capacity (0 GU) to the commercially available antimycobacterial drugs, streptomycin, isoniazid, rifampicin, and ethambutol (SIRE) (Table 3). These findings are consistent with other studies that have demonstrated that plants are an excellent potential source of active antitubercular compounds [18, 39, 51, 75]. However, solvent fractionation yielded relatively lower general antimicrobial activity against Gram-positive, Gram-negative, and fungi compared to the crude extract (Tables 2 and 4). This phenomenon suggests a possible synergistic and/or additive effect of the active molecules in the crude extract, an effect that was possibly lost by fractionation [76, 77, 78, 79]. Furthermore, the fact that fractions demonstrated a robust antitubercular activity, and low general activity against Gram-positive, Gram-negative, and fungi allude to a possible selective activity against MTB, which is an essential feature in search of novel selective antitubercular leads [69, 70].

Bioactivity of plant extracts is the work of secondary metabolites produced for purposes of normal plant defenses; to deter, stun, poison or kill threatening species, but inadvertently inhibiting various physiological targets/processes required for growth, biosynthesis of macromolecules, metabolism, and virulence of microbial systems [30, 80, 81, 82]. To characterize and identify the active compounds mediating activity against the tested organisms, we initially performed qualitative phytochemical screening in all extract solvent fractions (Table 6), and subsequent GC-MS analysis of the methanolic solvent extract fraction (Table 7 & Figures 1 and 2). We qualitatively identified terpenoids, phenolics such as flavonoids and anthraquinones, and alkaloids in *A. pluriseta* extracts, and thus speculated that they are the phytochemicals partly or wholly responsible of the antitubercular activity demonstrated by various extract fractions. Previous studies have also reported presence of terpenoids, alkaloid, flavonoids, anthraquinones and phenolic in *A. pluriseta* aqueous extract [40, 47, 83, 84]. Terpenes were enriched in all fractions tested, and a broad range of terpenes identified in other studies have been associated with antitubercular activity, partly due to their lipophilicity that makes it easier for them to penetrate through MTB wall [85, 86]. Alkaloids, terpenoids, and flavonoids have been speculated to induce antitubercular activity by interfering with the MTB efflux pump, thus modulating in non-specific manner membrane proteins and receptors as well as inhibiting natural methods for MTB resistance development [87].

Additionally, using GC-MS, we identified two abundant fatty acid alkyl esters in methanolic solvent extract fraction; 2-hydroxy-1-(hydroxymethyl) ethyl ester-hexadecanoic acid (2-palmitoylglycerol) and 2,3-dihydroxypropyl ester-octadecanoic acid (glyceryl monostearate); (Table 7 and Figures 1 and 2). We speculate that these fatty acid alkyl esters are associated with the observed bioactivity of this extract fraction. In fact, previous studies have reported antimicrobial and antimycobacterial activities of naturally occurring and synthetic fatty acid alkyl esters [88, 89, 90, 91]. Although the relationship between structure and antimicrobial activity of various fatty acids and their ester derivatives remains elusive, it is speculated that the inhibitory effect is greatly influenced by the number and presence of double bonds [90]. Mechanistically, fatty acids and their ester derivatives (FAED) act by interfering with the vital fatty acid synthase-I (FASI)- and FASIII-mediated fatty acid biosynthesis and degradation [92]. Furthermore, FAED and long-chain alkanes, by acting as exogenous mimics of microbial cell wall and membrane fatty acid derivatives, have been suggested to interfere with the cell membrane and wall biochemistry thus inducing an antimicrobial activity [93, 94, 95]. Damage to cell envelope leads to perturbation of ion homeostasis across the membrane, defective cell to cell adhesion, disturbed substrate attachment, and consequent inability to form biofilm, which is essential for tubercular pathogenicity [14]. However, it is necessary to isolate individual pure compounds and test their activity individually and/or in combination. Taken together, we show that *A. pluriseta* extract solvent fractions have robust selective antitubercular activity, and hence we provide a scientific rationalization and justification of the possible therapeutic use of *A. pluriseta*.

## 5 Conclusion

The findings from this work demonstrate that *A. pluriseta* root extract fractions have robust selective antitubercular activity. The extract fractions, especially the ethyl acetate and methanolic fraction, provide a potential source of novel, antitubercular lead candidate(s). GC-MS analysis revealed an abundance of fatty acid esters, which we strongly associated with demonstrated antitubercular activity. Further work is required to isolate pure compounds, test their specific molecular targets, with a view of deciphering the mode(s) of action.

## Declarations

### Author contribution statement

S.N. Njeru and J.M. Muema: Conceived and designed the experiments; Performed the experiments; Analyzed and interpreted the data; Contributed reagents, materials, analysis tools or data; Wrote the paper.

### Funding statement

This study was supported by the 10.13039/100004413International Foundation for Science, Stockholm, Sweden, through IFS grant No. F/5372-1 to Dr. Sospeter Ngoci, Njeru.

### Competing interest statement

The authors declare no conflict of interest.

### Additional information

No additional information is available for this paper.
